# Mouse Nuclear Myosin I Knock-Out Shows Interchangeability and Redundancy of Myosin Isoforms in the Cell Nucleus

**DOI:** 10.1371/journal.pone.0061406

**Published:** 2013-04-11

**Authors:** Tomáš Venit, Rastislav Dzijak, Alžběta Kalendová, Michal Kahle, Jana Rohožková, Volker Schmidt, Thomas Rülicke, Birgit Rathkolb, Wolfgang Hans, Alexander Bohla, Oliver Eickelberg, Tobias Stoeger, Eckhard Wolf, Ali Önder Yildirim, Valérie Gailus-Durner, Helmut Fuchs, Martin Hrabě de Angelis, Pavel Hozák

**Affiliations:** 1 Department of Biology of the Cell Nucleus, Institute of Molecular Genetics, ASCR, v.v.i., Prague, Czech Republic; 2 Institute of Laboratory Animal Science and Biomodels Austria, University of Veterinary Medicine Vienna, Vienna, Austria; 3 German Mouse Clinic, Institute of Experimental Genetics, Helmholtz Zentrum München, German Research Center for Environmental Health, Neuherberg/Munich, Germany; 4 German Mouse Clinic, Comprehensive Pneumology Center and Institute of Lung Biology and Disease, Helmholtz Zentrum München, German Research Center for Environmental Health, Neuherberg/Munich, Germany; 5 Chair of Molecular Animal Breeding and Biotechnology, Gene Center, Ludwig-Maximilians-Universität München, Munich, Germany; 6 Chair of Experimental Genetics, Center of Life and Food Sciences Weihenstephan, Technische Universität München, Freising-Weihenstephan, Germany; 7 Member of German Center for Diabetes Research, Neuherberg/Munich, Germany; 8 Faculty of Science, Charles University in Prague, Prague, Czech Republic; University of Massachusetts Medical, United States of America

## Abstract

**Background:**

Nuclear myosin I (NM1) is a nuclear isoform of the well-known “cytoplasmic” Myosin 1c protein (Myo1c). Located on the 11^th^ chromosome in mice, NM1 results from an alternative start of transcription of the Myo1c gene adding an extra 16 amino acids at the N-terminus. Previous studies revealed its roles in RNA Polymerase I and RNA Polymerase II transcription, chromatin remodeling, and chromosomal movements. Its nuclear localization signal is localized in the middle of the molecule and therefore directs both Myosin 1c isoforms to the nucleus.

**Methodology/Principal Findings:**

In order to trace specific functions of the NM1 isoform, we generated mice lacking the NM1 start codon without affecting the cytoplasmic Myo1c protein. Mutant mice were analyzed in a comprehensive phenotypic screen in cooperation with the German Mouse Clinic. Strikingly, no obvious phenotype related to previously described functions has been observed. However, we found minor changes in bone mineral density and the number and size of red blood cells in knock-out mice, which are most probably not related to previously described functions of NM1 in the nucleus. In Myo1c/NM1 depleted U2OS cells, the level of Pol I transcription was restored by overexpression of shRNA-resistant mouse Myo1c. Moreover, we found Myo1c interacting with Pol II. The ratio between Myo1c and NM1 proteins were similar in the nucleus and deletion of NM1 did not cause any compensatory overexpression of Myo1c protein.

**Conclusion/Significance:**

We observed that Myo1c can replace NM1 in its nuclear functions. Amount of both proteins is nearly equal and NM1 knock-out does not cause any compensatory overexpression of Myo1c. We therefore suggest that both isoforms can substitute each other in nuclear processes.

## Introduction

Myosins are unique proteins that have the ability to transform free chemical energy stored in ATP into mechanical force. In comparison to the well-known “conventional” class II myosins found in muscles, there is a variety of other “unconventional” myosins belonging to several groups. Myosin I family members are monomeric, non-processive, slow-rate and low-duty ratio molecular motors. Myosin 1c (Myo1c) was the first single-headed myosin isolated from mammals and it was therefore called mammalian myosin I [1], [2]. Based on its similarity to partial myosin sequence from mouse cDNA library, it was later renamed as myosin 1β [3], and finally, after the unification of myosin I nomenclature, myosin 1c [4]. The human MYOIC gene encodes three isoforms. Myosin 1c isoform C is the classic 1063 amino acid “cytoplasmic” form [2]. Myosin 1c isoform B, also known as nuclear myosin 1 (NM1), includes 16 extra N-terminal amino acids arising from an upstream exon -1 [5], [6]. The newest isoform is myosin 1c, isoform A, which includes additional 35 amino acids on its N-terminal end from an upstream exon -2 and was described to work in the cell nucleus [7]. In mice there have been only two myosin isoforms described – Myo1c and NM1.

Myosin 1c (isoform C) belongs to a group of molecular motors that link cellular membranes to the actin cytoskeleton, and are involved in membrane tension generation, membrane dynamics, and mechanosignal transduction. In detail, Myo1c was identified to be associated with Neph1 and nephrin proteins. Myo1c mediates their localization to the plasma membrane and its depletion causes defects in tight junctions' formation and cell migration [8]. In the neuronal growth cone, Myo1c affects lamellipodial motility and is responsible for retention of lamellipodia [9] and retrograde F-actin flow [10]. In *Xenopus laevis*, Myo1c participates in egg activation by coupling dynamic actin to the membranes of cortical granules, and this linkage is essential for their compression and retrieval [11]. Insulin stimulates glucose transport in adipocytes by promoting exocytosis of the population of vesicles containing glucose transporter protein GLUT4. Myo1c enhances exocytosis of GLUT4-containing vesicles to plasma membrane [12], [13]. The most extensively studied function of Myo1c concerns the process of hearing. Sensory cells of the inner ear detect sound and transmit signals representing those stimuli to the central nervous system. Myo1c has a direct function in the adaptation of sensory cells to sustained excitatory deflection, since upon its inhibition the adaptation is slow and inefficient [14]. Moreover, clinical studies revealed 6 missense mutations in Myo1c that were associated with bilateral hearing loss [15].

All these functions connect Myo1c to the plasma membrane and actin filaments. This was further proved by Nambiar et al. (2009) who showed that Myo1c together with other myosin I family members mediate membrane/cytoskeleton adhesion. This makes major contributions to membrane tension, which is one of the main parameters needed in endo- and exocytosis, membrane repair, cell motility, and cell spreading.

Nuclear myosin I (isoform B) was discovered coincidentally by testing of affinity-purified polyclonal antibodies to adrenal myosin 1. The antibody was staining a 120-kDa nuclear protein with ATPase activity, and ATP-, actin- and calmodulin- binding which are the typical features of unconventional myosins [5]. The mass spectrometric analysis of the immunopurified protein showed high homology to the Myo1c protein. Due to the fact that at that time, NM1 was the first myosin found in the cell nucleus, it was called nuclear myosin I [6]. In the nucleus, NM1 associates with nuclear actin and is required for RNA polymerase I (Pol I) and RNA polymerase II (Pol II) transcription [6], [16]. Both NM1 and actin co-localize and co-immunoprecipitate with Pol I and Pol II complexes. *In vitro* immunodepletion of NM1 inhibits transcription by both polymerases and the addition of purified NM1 increases the level of transcription in a dose-dependent manner. While both proteins associate with Pol I, actin associates with Pol I regardless of the transcriptional state. In contrast, NM1 only associates with initiation-competent RNA polymerase I complexes through an interaction with the basal transcription factor TIF1A [16]. In addition to transcription initiation, NM1 is needed in further steps during elongation phase where it interacts with chromatin remodeling complex WSTF-SNF2h and facilitates Pol I transcription on chromatin [17]. It is therefore believed that NM1 bound to TIF-1A is recruited to the pre-initiation complex along with Pol I and associated actin to assemble a functional transcription initiation complex. Recruitment of Pol I to the NM1-TIF-1A complex might facilitate the interaction of NM1 with actin bound to Pol I. Finally, by interacting with NM1, chromatin remodeling complexes join the initiation complex to promote Pol I movement through chromatin [18]. This is also supported by the finding that both actin polymerization and the motor function of NM1 are required for association with the Pol I transcription machinery and transcription activation [19]. Moreover, NM1 was found in interaction with RNA and RNA-protein complexes present in the nucleoplasm and in nucleoli [20]. It participates in the maturation of pre-rRNA, and accompanies rRNA transcripts to the nuclear pore where NM1 decorates actin-rich pore-linked filaments [21]. Aside from its functions in transcription, Chuang et al. (2006) showed that the actin-NM1 complex is needed for long-range directional movement of interphase chromosome sites independently from their engagement in transcription. NM1 is also able to bind DNA directly via its tail domain [22], and NM1 together with gelsolin were identified as key determinants for assembling and/or stabilization of complexes containing estrogen receptor α (ERα) and actin in the nucleus early after receptor activation by its ligands [23].

Both Myo1c and NM1 proteins are shown to be expressed in a wide variety of tissues and cultured cell lines. Their expression pattern is similar but not completely overlapping. Both proteins have the highest level of expression in mouse lungs, followed by intestine, kidney, heart and spleen for NM1, and adrenal gland, stomach, spleen, heart and esophagus for Myo1c. These expression profiles suggested possible tissue-specific functions for both proteins [2], [24], [25]. However, Myo1c is localized mostly at the cell periphery and at the plasma membrane, particularly at the leading edges of motile cells [2], while NM1 was described to localize mostly in the nucleus where its distribution is dependent on transcriptional activity of the cell [26], [27]. The 16 amino acid N-terminal extension of NM1 molecule was thought to be the nuclear localization signal for this protein [6]. However, we recently showed that nuclear localization signal which triggers NM1 to the nucleus is located in the middle part of the molecule and therefore direct both NM1 and Myo1c to the nucleus. This novel calmodulin-dependent NLS is localized in the second of three IQ domains [24]. Because of the various nuclear functions of NM1, it is important to determine if both isoforms can perform identical functions. We therefore prepared mice lacking NM1 isoform without affecting Myo1c expression and here we describe observed phenotypes in NM1 knock-out mice. Surprisingly, these mice were fully viable and did not show any obvious defects related to previously described functions of NM1 in DNA transcription. Moreover, the ratio between both isoforms in the nucleus and cytoplasm is roughly equal. We therefore tested Myo1c's capability to act in transcription. We found that Myo1c is able to functionally substitute NM1 in Pol I transcription. Moreover, it directly binds to the Pol II CTD domain suggesting it has also a role in Pol II transcription. In conclusion, we suggest that the two isoforms Myo1c and NM1 (isoforms C and B) are mutually redundant in general process of transcription.

## Materials and Methods

### Ethics statement

All animal experiments and work with human and mice cell lines conformed to relevant regulatory standards and were approved by the Ministry of Agriculture of the Czech Republic, the ethic committee of the Institute of Molecular Genetics ASCR (animal experiment license no. 40/2009 and 186/2010) and the district government of Upper Bavaria (Regierung von Oberbayern).

The project for the generation of the mutants was discussed and approved by the institutional ethics committee of the Vedmeduni Vienna and animal experiment license granted under no. BMWF-68.205/0084-II/10b/2008. All animal experiments performed in GMC were done with permissions of the appropriate authorities according to the §8 of the German Law for the Protection of Animals.

### Plasmids

NM1-Fl and Myo1c-Fl vectors were prepared by cloning of NM1 and Myo1c cDNA into pCDH-CMV-MCS-EF1-Neo (Systems Bio) using SnaBI and EcoRI restriction enzymes in frame with the C-terminal Flag tag. The constructs expressing shRNA-resistant NM1-GFP and Myo1c-GFP was prepared by excision of mouse NM1-GFP and Myo1c-GFP from vectors NM1-GFP and Myo1c-GFP [24] and ligated into pCDH-EF1-Neo (Systems Bio). The NM1-GFP construct has been previously described [24].

### Preparation of targeting construct for knock-out mice

**Figure 1:**
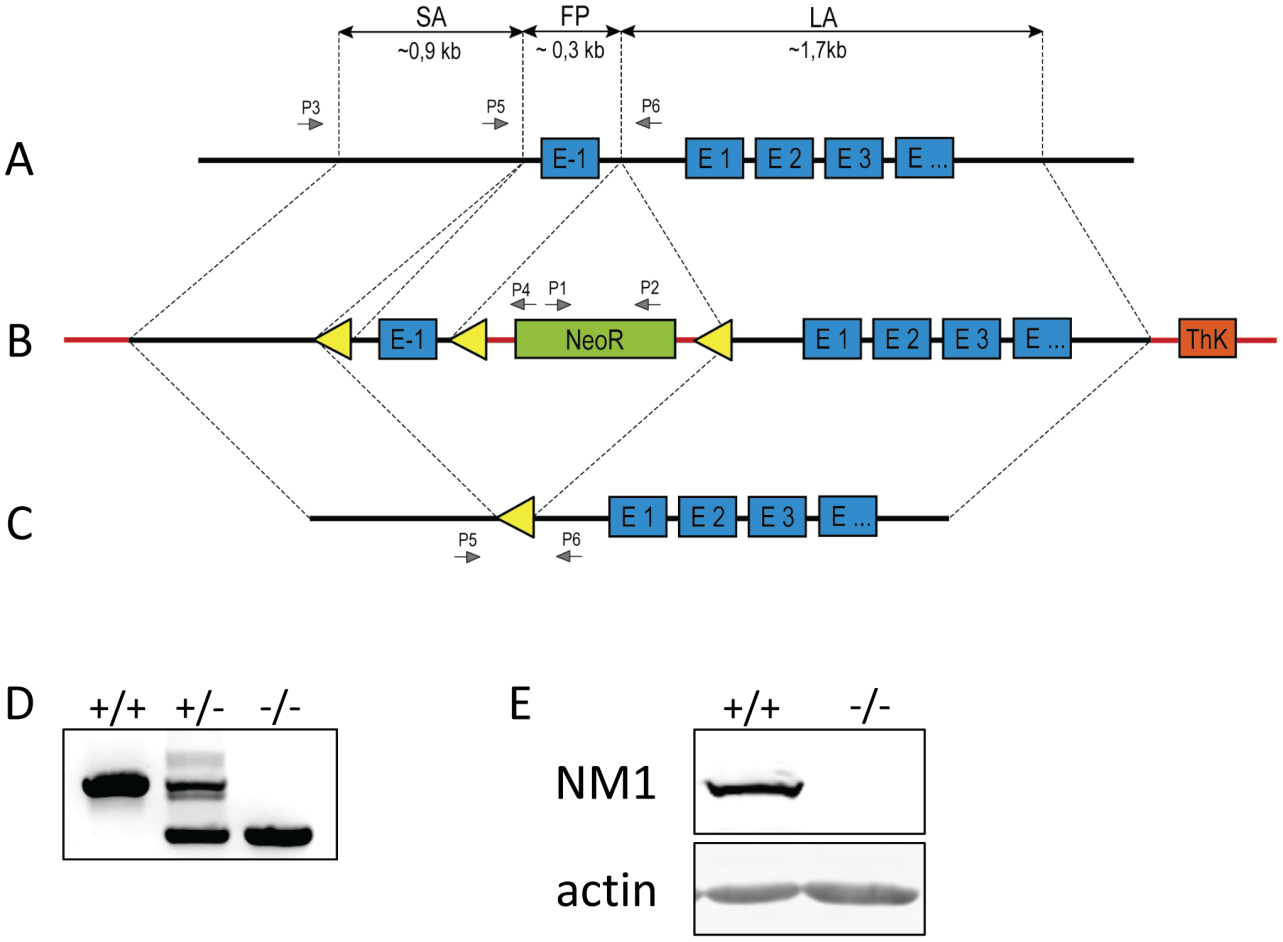
Preparation of NM1 knock-out cassette. (**A**) WT genomic locus of *Myo1c* gene. Short homology arm (SA), floxed part (FP), and long homology arm (LA) of appropriate length (0.9; 0.3; 1.7 kb respectively), were cloned to pEasyFlox vector carrying neomycin (NeoR) and thymidine kinase (ThK) selection genes (**B**). Black lines represent genomic sequences; red line represents sequences derived from pEasyFlox vector. (**B**). (**C**) Genomic loci of *Myo1c* gene with excision of exon -1; P1 – P6 represent different primers needed for genotyping of ES cells and knock-out mice, yellow triangles represent loxP recombination sites. (**D**) Genotyping of NM1 knock-out mice by PCR. P5 and P6 primers were used to distinguish between wild type (+/+), heterozygous (+/−) and knock-out (−/−) animals. (**E**) Western blot analysis of NM1 levels in NM1 wild type (+/+) and knock-out (−/−) mice. Fifteen micrograms of protein per lane was loaded, and probed for NM1. Equal loading was monitored by Coomassie Brilliant Blue staining of the band corresponding to actin.

For the preparation of conditional NM1 knock-out (KO), the Cre–loxP system and a targeting construct based on pEasyFlox vector was used [28].The short homology arm (SA,∼0,9 kb) was prepared by PCR amplification of genomic DNA sequence from -1096 to -166 base pairs from NM1 translation initiation site using primers SA F (5′-gtagagtcgacTATGCCACAAGAGGTGGCAACT-3′) and SA R (5′-gccgaagcttCCGGGCTGGGTGGGAGGGGGTTCG-3′). The long homology arm (LA,∼1,7 kb) was prepared by amplification of genomic DNA from +116 to +1800 base pairs from the NM1 translation initiation site, using primers LA F (5′-ggggatccGGTGGAAGATGTCCCTGAAAGTTG-3′) and LA R (5′-gctgcggccgcGTAGTAACCTGGGCATTGCTGTCC-3′). The floxed part encoding the -1 exon (containing the NM1 start codon); (FP,∼0,3 kb) was prepared by amplification of genomic DNA from -165 to +115 base pairs from the NM1 translation initiation site, using primers FP F (5′-cccagtcgacCCAGGCCGGCTGCAGTGGGTCCTA-3′) and FP R (5′-catcttctagaGAAATTCCTGGGCCGCGCCCGCTT-3′) (**Fig.1A**). All PCR reactions were done using mouse 129/Sv genomic DNA as a template. The SA PCR product was digested and cloned into the pEasyFlox vector via XhoI and HindIII restriction sites, LA by BamHI and NotI and FP via SalI and XbaI restriction sites. For screening of positively electroporated cells with the targeting construct, we used a neomycin-resistance positive selection marker (Neo) flanked with loxP sites (**Fig. 1B**).

### Generation of NM1 knock-out mice

To test the influence of the N-terminal 16 amino acids on NM1 functions, we generated mice lacking exon -1 of *Myo1c* which contains the NM1 start codon [6]. Exon -1, and both the long and the short arm were cloned into the pEasyFlox vector carrying neomycin resistance and thymidyne kinase selection cassettes together with three loxP sites (Fig 1A, 1B, 1C). The linearized NM1 tri-loxP construct was then electroporated into the TBV2 (129S2/SvPas) ES cell line (provided by T. Rülicke) as follows: TBV2 ES cells were cultured in ES cell medium (DMEM with 4.5 g/L glucose, 15% FBS, 1 mM Sodium-pyruvate, 2 mM glutamine, 1x NEAA, 1x penicillin/streptomycin, 0.1 mM 2-mercaptoethanol and 1000 µ/ml pure leukemia inhibitory factor (Lif)). All ingredients except Lif (Millipore) and 2-mercaptoethanol (Sigma) were purchased from PAA. ES cells were grown on 6 µcm plates coated with inactivated mouse embryonic fibroblasts (MEF). Ten plates were then pooled and re-suspended in 1 µml PBS. About 2×10^7^ cells were mixed with 30 µg linearized *Myo1c* targeting construct and chilled on ice before electroporating in a 0.4 µcm electroporation cuvette using a Gene Pulser Xcell (BioRad); (settings 500 µF and 0.23 kV). Electroporated cells were plated onto irradiated neo-resistant MEF cells on ten 6 µcm plates. Selection with 300 µg/ml G-418 sulphate (PAA) was started 24 µh after electroporation. Neo-resistant colonies were picked after 14 days of G-418 treatment and screened by PCR. PCR-positive clones were confirmed for correct targeting by Southern blot analysis and karyotyped for euploidy. One clone was selected for injection into C57BL/6N blastocysts.

The NM1 tri-loxP conditional mutation (**Fig. 1B**) was induced in the 129/S2 genetic background. Male chimeras with more than 50% ES-derived coat color were bred with C57BL/6NCrl female mice to test for germ-line transmission and the resulting litters with appropriate coat color were genotyped by PCR for the targeted mutation. Heterozygous mutants carrying the NM1 tri-loxP allele were mated with MeuCre40 transgenic mice on C57BL/6N background in order to excise the floxed neo and exon -1 cassettes [29]. The resulting offspring were screened for the presence of Cre and for the partial recombination of the NM1 tri-lox allele by PCR (**Table 1**). Heterozygous NM1 knock-outs with a partial deletion of either only the neo cassette or the complete deletion of both exon -1 and the neo cassette were back-crossed to C57BL/6N (Charles River Laboratories, Sulzfeld, Germany) for 5 generations. The resulting mutants were designated as B6;129S2-*Myo1c^tm1(flox)^* ^Biat^ for the conditional allele and B6;129S2-*Myo1c^tm1.1^* ^Biat^ for the constitutive NM1 knock-out. For all further experiments, mutants (KO) and wild type (WT) controls were produced by mating heterozygous NM1 knock-out mice and their final genotype was proven either by PCR (**Fig. 1D**) or western blot with antibody specific to N-terminal domain (**Fig. 1E**). Mice were housed under standard conditions, (mean room temperature 21+/−1° Celsius, 40–55% relative humidity, 12∶12h light-dark cycle) and supplied with standard breeding diet (ssniff Spezialdiäten GmbH, V1126, Germany) and tap water *ad libitum.* Depending on the experiment, all mice used for analysis were aged between 3 to 15 months.

**Table 1:** PCR primers for genotyping of ES cells and mice mutants.

Name	Sequence 5′-3′
P1	AGACAATCGGCTGCTCTGAT
P2	CTCGTCCTGCAGTTCATTCA
P3	GGGTAGTAGTGGTGTTGATGGCTTGG
P4	TGTTCCACATACACTTCATTCTCAG
P5	TTCCTCCTGGAAAACCTGACTC
P6	CTCTGCTTCTCCGTCACCC
CreF	CTGGAAAATGCTTCTGTCCG
CreR	CAGGGTGTTATAAGCAATCCC

### Genotyping of NM1 knock-out mice

Genotypes of ES cells and mice were confirmed by PCR of genomic DNA using consecutive primers. For identification of targeted ES cells, P1 and P2 primers inside the neomycin-resistance gene were used. Homologically recombined targeting constructs were identified with P3 and P4 primers. The presence of Cre recombinase was shown by CreF and CreR primers. Partial recombination of the NM1 tri-lox allele, together with recognition of full NM1 KO, WT and heterozygous mice was done by P5 and P6 specific primers. All primers with sequences are summarized in **Table 1**.

### Cell culture and lentivirus work

Primary skin fibroblasts were isolated from ear explants. Skin samples were washed in PBS and incubated with 0,3% trypsin/PBS for 60 min in a 37°C water bath. Samples were then cut into small pieces, placed on the bottom of Petri dish, overlaid by sterile glass coverslip and supported by complete growth DMEM medium with 10% FBS. Medium was changed every 3 days until cells grew to confluence. Cells were held for a maximum of 10 passages. Stable cell lines were prepared by long-running cultivation of primary cell culture (over 20 passages). All cell types were grown in humidified 5% CO_2_/air, 37°C environment.

For testing of interchangeability of the two myosin isoforms, stable knock-down of human NM1 and Myo1c with exogenous expression of mouse NM1 or Myo1c in U2OS cell line (ATCC No. HTB-96) was prepared. U2OS cells were transduced by pCDH-EF1-Neo carrying mouse Myo1c or NM1 and by pLKO1.1 vector expressing shRNA targeting the sequence 5′ -GCCCGTCCAGTATTTCAACAA- 3′ (Open Biosystems cat No TRCN0000122925 AAO75-C-8). U2OS cells either stably expressing mouse Myo1c-GFP, NM1-GFP or expressing only the endogenous human protein were transduced with recombinant lentiviruses expressing shRNA targeting only human cDNA. 3 days post transduction Pol I transcription rates were compared in WT U2OS cells, U2OS cells expressing shRNA targeting human NM1 and Myo1c and U2OS cells expressing shRNA with exogenous expression of Myo1c-GFP or NM1-GFP by using quantitative PCR. Recombinant lentiviruses were prepared using second generation viral packaging system (Didier Trono Lab).

For immunoprecipitation experiments, a human H1299 cell line (ATCC No. CRL-5803) stably expressing either NM1-Fl or Myo1c-Fl was prepared by lentivirus transduction of pCDH-CMV-MCS-EF1-Neo vectors carrying NM1-Fl or Myo1c-Fl constructs.

HeLa cells (ATCC No. CCL-2) were used for defining the protein ratio of the two isoforms in the cell nucleus and cytoplasm.

### Antibodies

For western blots and immunofluorescence detection of NM1, rabbit polyclonal antibody specific to N-terminal part of NM1 (M3456, Sigma) or rabbit polyclonal anti-NM1 antibody, which was kindly provided by Piergiorgio Percipalle [20] were used. Polyclonal antibody (R2652) against the tail domain of Myo1c was kindly supplied by Peter G. Gillespie, Oregon Hearing Research Center and Vollum Institute [30]. All other antibodies used in this study are commercially available: antibody against β-actin (A2066) was purchased from Sigma, anti-GAPDH antibody (clone 6G5) is available from Acris antibodies, anti-FLAG tag antibody (200471) from Stratagene, and anti-RNA polymerase II CTD phospho S2 (H5; ab24758) from Abcam.

### Quantification of NM1/Myo1c ratio using LI-COR Odyssey© system

To explore the ratio between both isoforms in the cell nucleus and cytoplasm in HeLa cells, LI-COR Odyssey© fluorescent detection system was used. A polyclonal antibody to the N-terminus and a monoclonal antibody that was generated against the tail domain of NM1/Myo1c (described above) were coupled to infrared dyes IRDye 680 and IRDye® 800CW (LI-COR), which enables quantification of fluorescent signal in two separate channels. To normalize the signal from the two antibodies, we transfected cells with a NM1-GFP construct that has the molecular weight of 170 kDa.

### Immunoprecipitation of NM1-Fl and Myo1c-Fl

H1299 cell line stably expressing NM1-Flag or Myo1c-Flag was prepared by lentivirus transduction. For the experiment cells were washed with PBS and extracted with buffer containing 50 mM HEPES pH 8, 300 mM NaCl, 4 mM MgCl_2_ and 1% Triton X-100 and sonicated. Extract was cleared by centrifugation and filtered through 0.45 µm filter. Clear lysate was incubated 2 hours with 15 µl of pre-equilibrated Flag-M2 agarose (Sigma). As a control, lysates were incubated 2 hours with control IgG from pre-immune serum cross-linked to protein G-agarose beads. After the incubation beads were washed 3 times with buffer containing 50 mM HEPES pH 8, 300 mM NaCl and 4 mM MgCl_2_. Finally, the bound proteins were boiled for 5 min into SDS-PAGE loading buffer and analyzed by SDS-PAGE.

### Phenotyping of mice

A broad phenotype analysis of 50 wild type littermates as controls (29 males, 21 females) and 46 NM1 knock-out mice (27 males, 19 females) was done in collaboration with the German Mouse Clinic (Helmholtz Zentrum München-Deutsches Forschungszentrum für Gesundheit und Umwelt (GmbH), Neuherberg, Germany). Mice, 9–18 weeks old, were analyzed for irregularities in dysmorphology, behavior, neurology, nociception, eye function, energy metabolism, clinical chemistry and hematology, immunology, allergy reaction, steroid metabolism, cardiovascular and lung function, and pathology according to standardized protocols [31], [32].

#### Bone density analysis

After anesthesia, the weight and length of the mouse were recorded, and the mouse was placed in the pDEXA Sabre X-ray Bone Densitometer (Norland Medical Systems. Inc., Basingstoke, Hampshire, UK). After a scout scan, the area of interest was optimized and the measurement scan started using following settings: scan speed 20 mm/s, resolution 0.5 mm×1.0 mm, and HAW 0.020. The standard analysis comprises a whole body analysis as well as a whole body analysis excluding the skull. Analysis of quantitative data sets was carried out using StatView software package (SAS Corporation).

#### Hematology analysis

To investigate the peripheral blood cell count, a blood volume of about 50 µl EDTA-blood was used to measure basic hematological parameters with a blood analyzer, which has been validated for the analysis of mouse blood using the laboratory mouse chip card (ABC-Blutbild-Analyzer, Scil Animal Care Company GmbH; Viernheim, Germany). Number and size of red blood cells were measured by electrical impedance, and hemoglobin by spectrophotometry. Mean corpuscular volume (MCV) was calculated directly from the cell volume measurements. The hematocrit (HCT) was assessed by multiplying the MCV with the red blood cell count. Mean corpuscular hemoglobin (MCH) and mean corpuscular hemoglobin concentration (MCHC) were calculated from hemoglobin/red blood cell count and hemoglobin/hematocrit respectively. Data were statistically analyzed using an R-Script, applying ANOVA (testing effects of genotype, sex and the interaction of both) and subsequent pair-wise comparisons using the Tukey *post hoc* test, and the Wilcoxon Rank Sum Test on genotype differences separately for each sex and over all, with the level of significance set at p<0.05.

#### Pulmonary function analysis

All mice were anesthetized by i. p. injection of ketamine (137 mg/kg body weight) and xylazin (6.6 mg/kg body weight). After opening the trachea by a small incision, an 18 gauge cannula was inserted and fixated by ligation. The mice were then placed in a FinePointe RC system (Buxco Research Systems; Wilmington, NC, USA). In a heated plethysmograph chamber, mice were ventilated at an average rate of 160 breaths per minute, and flow and mouth pressure and heart rate were monitored to measure resistance and dynamic compliance. Beginning with an initial acclimation period of three minutes, a two-minute measurement was performed. After the resistance (R) and compliance (Cdyn) measures, mice were transferred to a forced pulmonary maneuvers system (Buxco Research Systems; Wilmington, NC, USA). Here, the forced residual capacity (FRC) was determined during spontaneous breathing of the mouse using Boyle's Law, then quasistatic pressure volume (PV) and fast flow volume (FV) maneuvers were run three times each and averaged to obtain all lung volume and flow parameters. The PV test uses a slow expiration phase after inflating the mouse to total lung capacity (TLC) to obtain quasistatic chord compliance (Cchord), TLC, expiratory reserve volume (ERV), residual volume (RV) and inspiratory capacity (IC). The FV test applies a fast expiration after inflation to TLC, thereby measuring forced vital capacity (FVC), peak expiratory flow (PEF) and forced expiratory volume at 100 ms (FEV100).

Measurements were always performed between 8 a.m. and 1 p.m. The system was set up in a quiet room where temperature and humidity were kept constant throughout the measurements. Statistical analyses were performed using R-scripts implemented in the database (MausDB). Differences between genotypes were evaluated by Wilcoxon test. Statistical significance was assumed at p<0.05. Data are presented as mean values±standard deviation (SD).

### Cell proliferation rate

Cells were seeded on a 6-well culture plate at a 20% confluence and left to grow for six days in full DMEM medium, and then the number of cells was counted. For quantitative analysis of cell viability and cell proliferation, we also used the redox indicator dye alamarBlue® (life technologies), which yields a colorimetric change in a response to a metabolic activity. For all the experiments primary as well as stable skin fibroblast derived from mice ear explants were used.

### RNA isolation and RT-qPCR

Total RNA from cells was isolated with GeneElute Mammalian total RNA Miniprep Kit (Sigma) according to manufacturer's protocol. The cells were lysed directly on cultivation plates. Concentration of RNA was measured by spectrophotometry and the integrity of RNA was checked on an agarose gel. 50 ng of total RNA was reverse transcribed and single-step real-time qPCR was performed with TaqMan® Reverse Transcription Reagents and PowerSYBR® Green PCR Master Mix (Applied Biosystems, Roche). RT-qPCR was performed in ABI Prism 7300 instrument (Applied Biosystems). For detection of mRNA of Myo1c and 45S pre-rRNA and GAPDH genes, the following primers were used: Myo1c CGATCACCCGAAGAACCA and GCGCTCTCCATGGTCACT, 45S pre-rRNA GGAGTGGGGGGTGGCCGG and GGGGAGAGGAGCAGACGAG, and GAPDH GGAAGGGCTCATGACCACAG and GCCATCCACAGTCTTCTGGG. Expression levels of 45S pre-rRNA and Myo1C were evaluated relative to GAPDH expression level.

## Results

### NM1 knock-out mice are viable and fertile

Because of the different expression pattern of NM1 in different tissues and the important roles of this protein in transcription [6], [16], chromatin remodeling [17], and chromosome movements [33], one would assume that NM1 knock-out would have lethal effect early during embryonic development or would have severe developmental defects. However, NM1 KO mice were fully viable with no significant differences from WT mice. They had a normal number of littermates and sex rate. 320 offspring from heterozygous mating have been checked: there were 157 males (49%) and 163 females (51%); 83 animals were wild type (25.9%), 159 heterozygous (49.7%) and 78 animals homozygous mutants (24.3%); the mean litter size was 6.09 pups per litter. These data indicate no major deviations from the expected numbers according to the Mendelian laws suggesting normal fertility of the heterozygous mice and viability of the homozygous mutants.

NM1 knock-out mice did not show significant differences in weight, size or physical condition, and haveńt shown any obvious behavior deviations or defects, or any pathology differences (data not shown). 50 wild type controls (29 males, 21 females) and 46 NM1 −/− mice (27 males, 19 females) have been analyzed for irregularities in dysmorphology, behavior, neurology, nociception, eye function, energy metabolism, clinical chemistry and hematology, immunology, allergy reaction, steroid metabolism, cardiovascular and lung function, and pathology in a comprehensive phenotypic screen in cooperation with the German Mouse Clinic.

In previous experiments, Kahle et al. (2007) have determined the expression profile of NM1 in different mouse tissues, with the highest NM1 protein levels in the lungs. Therefore, first we evaluated the qualitative aspects of lung function. Several parameters have been tested in female NM1 −/− mutants and then compared to wild type littermates (Tidal Volume, Inspiratory Capacity, Expiratory Reserve Volume, Vital Capacity, Functional Residual Capacity, Total Lung Capacity, Forced Vital Capacity, Flow Parameters, Forced Expiratory Volume, Peak Expiratory Flow, Static Lung Compliance, Dynamic Lung Compliance, and Lung Resistance). However, no significant genotype-specific differences in volumetric, flow or mechanical lung function parameters were found (**Table 2**). Therefore, despite high NM1 expression in lungs, the NM1 knock-out has no significant effect on pulmonary function *per se*.

**Table 2:** Lung function parameters tested in NM1 knock-out and wild type mice.

Parameter		Female WT	Female KO	p-value
		n = 6	n = 6	
Body Mass	[g]	28.2±2.5	26.1±3.3	0.223
**Volumetric Parameters**				
Tidal Volume	[ml]	0.203±0.005	0.206±0.007	0.665
Inspiratory Capacity	[ml]	0.88±0.087	0.841±0.119	0.394
Expiratory Reserve Volume	[ml]	0.27±0.06	0.3±0.04	0.294
Vital Capacity	[ml]	1.15±0.13	1.14±0.16	0.851
Functional Residual Capacity	[ml]	0.257±0.028	0.266±0.027	0.316
Total Lung Capacity	[ml]	1.137±0.094	1.107±0.131	0.394
Forced Vital Capacity	[ml]	1.052±0.102	1.028±0.156	0.394
**Flow Parameters**				
Forced Expiratory Volume	[ml]	1.024±0.095	0.999±0.151	0.416
Peak Expiratory Flow	[ml/sec]	29.9±0.6	28.5±1.7	0.197
**Mechanical Parameters**				
Static Lung Compliance	[ml/cm H2O]	0.0694±0.0084	0.0678±0.0104	0.732
Dynamic Lung Compliance	[ml/cm H2O]	0.0262±0.0028	0.0278±0.0042	0.818
Lung Resistance (R)	[cm H2O/ml/sec]	1.35±0.08	1.31±0.09	0.563

Further tests on NM1 knock-out evaluating deviations or pathological changes in different screens described above did not show any significant change between NM1 WT and KO animals (data will be given upon request).

**Figure 2:**
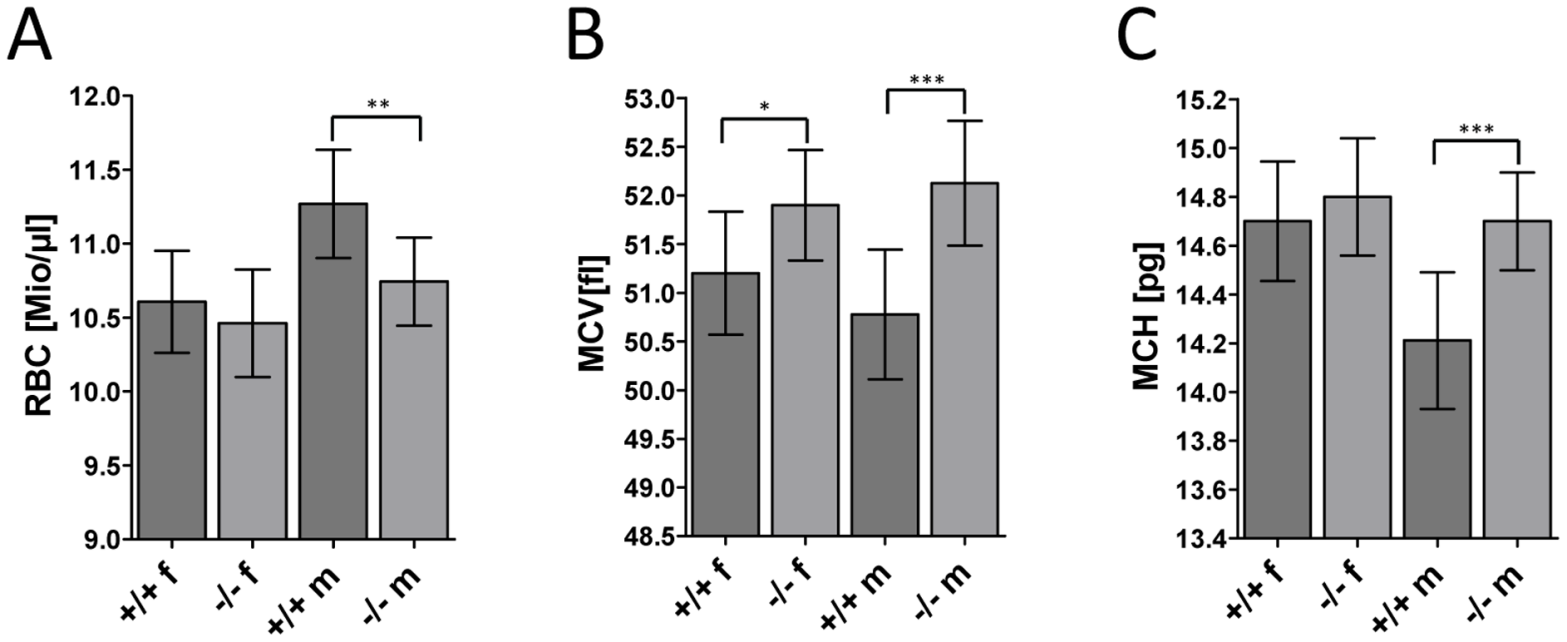
Red blood cells related phenotype in NM1 knock-out mice.

One group of tests evaluated bone and weight-related quantitative parameters of mature mice at the age of 14 weeks. Here NM1 −/− male mice had significantly (p ≤ 0.05 without correction due to multiple testing) increased bone mineral density in comparison to wild type mice (+/+: 45±4 mg/cm^2^, n = 10; −/−: 51±5 mg/cm^2^, n = 9). Other parameters such as bone mineral content, body weight, body length, fat and lean mass showed no significant change or deviation in comparison to the control littermates (**Table 3**). Finally, hematology screening results indicated mild macrocytosis in mutant animals associated with a trend towards an increased hemoglobin content in erythrocytes, which was accompanied by slightly reduced red blood cell counts and corpuscular hemoglobin concentration in males. Number of red blood cells was slightly decreased (**Fig. 2A**), the mean corpuscular volume (**Fig. 2B**) and mean corpuscular hemoglobin (**Fig. 2C**) were significantly increased. This observation might indicate impaired cell division during erythropoiesis [34].

**Table 3:** Overall body and bone parameters in NM1 knock-out and wild type mice.

Parameter		Male WT	Male KO	p-value
		n = 10	n = 9	
**Bone mineral density**	[mg/c∧2]	45±4	51↑±5	0.01
Bone mineral content	[mg]	350±95	286±86	0.147
Bone content	[%]	1.24±0.31	1.01±0.31	0.134
Body length	[cm]	9.68±0.22	9.70±0.18	0.835
Body weight	[g]	28.07±1.34	28.28±0.93	0.703
Fat Mass	[g]	2.73±2.01	2.13±1.28	0.459
Lean Mass	[g]	19.59±2.43	20.22±2.15	0.564

Taken together, these data show that knock-out of NM1 gene has no effect on mice viability and fertility, and broad phenotyping uncovered some minor but significant changes in bone mineral density and red blood cells parameters without any obvious pathological defects during mice proceeding.

### NM1 knock-out has no effect on cell viability, proliferation and transcription activity

**Figure 3:**
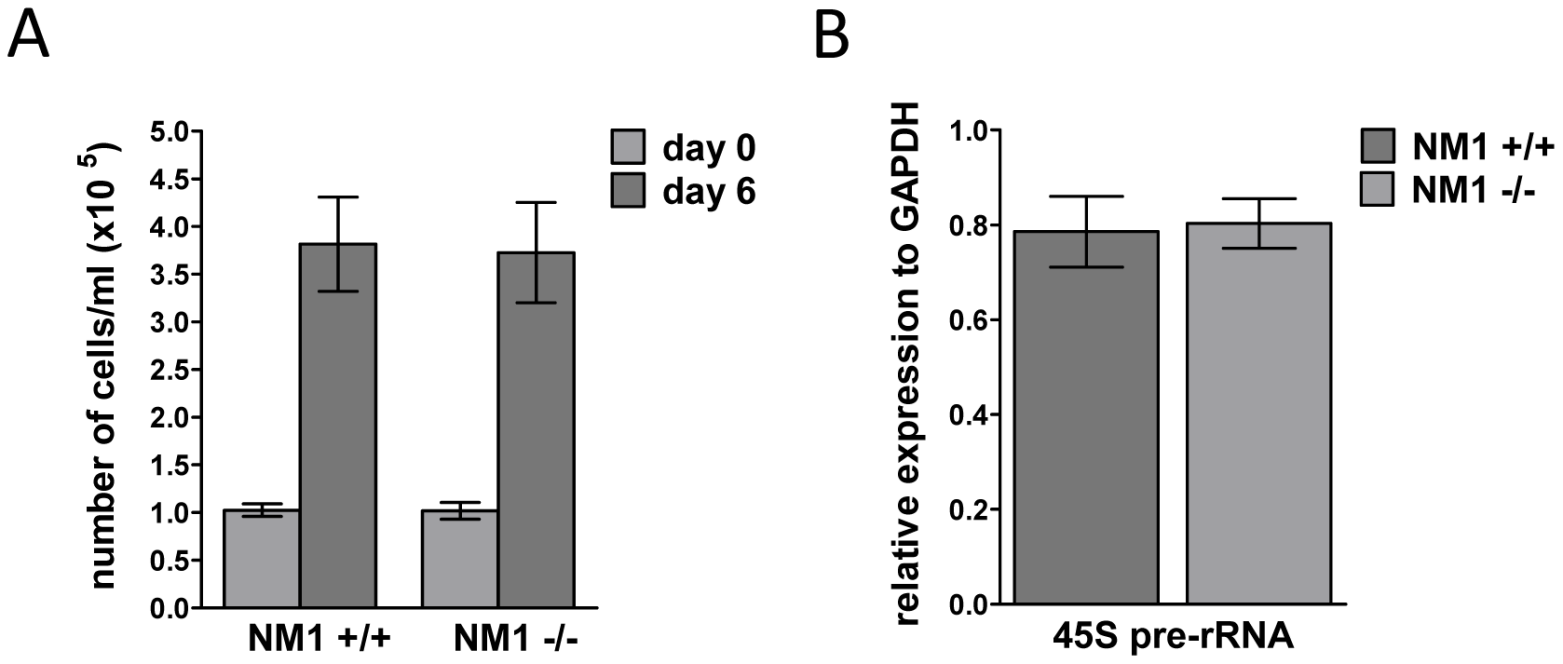
NM1 knock-out has no effect on cell proliferation and Pol I transcription. (**A**) Proliferation of NM1 KO cells (NM1 −/−) is not altered in comparison to WT cells (NM1 +/+). 1×10^5^ cells were seeded on plates (20% confluence; day 0) and let to grow for six days when number of cells was counted again (day 6). (**B**) Pol I transcription rate in NM1 KO (NM1 −/−) and WT (NM1 +/+) cells is equal. RNA from exponentially growing cells was isolated and expression of 45S pre-rRNA was measured by RT-qPCR. Expression of 45S pre-rRNA is compared relatively to GAPDH expression. Data are presented as mean +/− SD.

For further experiments, we prepared primary and stable fibroblast cell lines from NM1 KO and WT mice skin explants. These cell lines were used for evaluation of influence of NM1 deficiency at the cellular level. Typically, the condition of a cell highly correlates with the level of transcription activity. Therefore, we examined cell proliferation and viability by measuring the number of cells at different time points after seeding. After six days of growing, numbers of NM1 +/+ and NM1 −/− cells were the same, suggesting that cell proliferation and viability in NM1 KO cells was not affected (**Fig. 3A**). We also performed alamarBlue® assay to measure proliferation rate by colorimetric change in a response to a metabolic activity of the cells. The data re-confirmed the observations from the proliferation assay (data not shown).

Because of described functions of NM1 in RNA Pol I transcription, we directly explored whether NM1 knock-out has some effect on this process. We isolated RNA from NM1 WT and KO skin fibroblasts in the exponential growth phase, and measured the overall level of 45S pre-rRNA expression by RT-qPCR. NM1 knock-out had no effect on RNA Pol I transcription (**Fig. 3B**), and the expression level of 45S pre-rRNA in both cell lines was comparable.

Considering the connection between NM1 functions in Pol I transcription and the results described above, we can suggest that the function of NM1 protein in knock-out mice and cell lines is supplemented by functioning of other myosin protein or proteins.

### NM1/Myo1c expression is nearly equal in tissues and cell lines

We have previously shown that Myo1c protein differs from NM1 in just the first 16 amino acids [6]. Both proteins contain an identical NLS and both are able to enter the nucleus [24]. We therefore asked whether Myo1c would be able to substitute for the nucleus-related functions of NM1.

**Figure 4:**
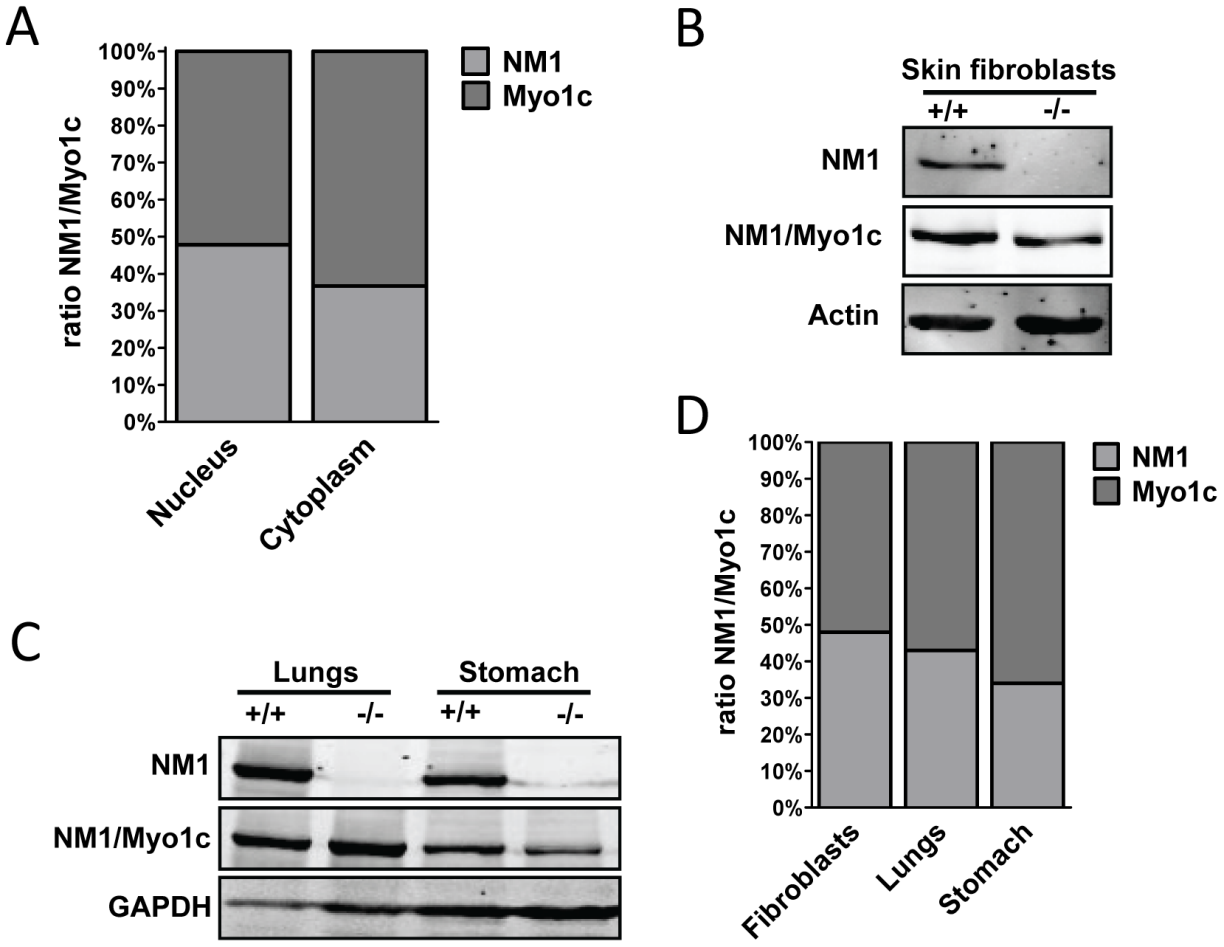
Ratio between NM1 and Myo1c is nearby equal. (**A**) HeLa cells were fractionated into cytosolic and nuclear fractions. NM1 and Myo1c amounts were quantified using double fluorescent labeling of western blot membranes after normalization to NM1-GFP band. (**B**) Total amounts of NM1+Myo1c were compared in mouse skin fibroblasts derived from NM1 knock-out and NM1 wild type mouse. Beta actin signal was used as loading normalizer. (**C**) Total amounts of NM1+Myo1c were compared in lungs and stomach from NM1 knock out and NM1 wild type mouse. GAPDH signal was used as loading normalizer. (**D**) The graph shows the amount of NM1 and Myo1c after densitometric quantification of bands from figures 4B and 4C showing the ratio between NM1 and Myo1c as compared to actin/GAPDH expression.

Firstly, we explored the ratio between both isoforms in the cell nucleus and cytoplasm in HeLa cells. Because the molecular weight of NM1 and Myo1c are very close to each other, we were unable to separate the two isoforms efficiently enough to quantify their amounts directly on western blots. We therefore used LI-COR Odyssey© fluorescent detection system that enables quantification of fluorescent signal in two separate channels. The intensities of NM1 and Myo1c were compared in the cytosolic and nuclear extracts of HeLa cells. We found that in this cell line, the ratio between NM1 and Myo1c was nearly 1∶1 in both cellular compartments (**Fig. 4A**). To confirm this result, we used NM1 knock-out skin fibroblasts in which the expression of NM1 is ablated and only Myo1c is expressed. We compared the level of total protein NM1+Myo1c using western blot. In KO cells, we observed about 50% reduction of signal intensity as compared to the wild type cells **(** **Fig. 4B** **)**. Similar reduction in fluorescent signal was observed using immunofluorescent labeling with an antibody against NM1/Myo1c (data not shown). We also repeated the quantification of NM1 and Myo1c using lungs and stomach tissues from NM1 KO in comparison to WT ones. Here the ratio of NM1 versus Myo1c was shifted more toward the Myo1c (∼55% in lungs, ∼64% in stomach tissue); (**Fig. 4C**).

Taken together, in cell types such as the primary fibroblasts and immortal cell lines, the NM1 isoform comprises about 50% of total NM1+Myo1c, while the ratio appears to be shifted toward the Myo1c isoform in mouse lungs and stomach (**Fig. 4D**).

### Myo1c is able to functionally substitute NM1

**Figure 5:**
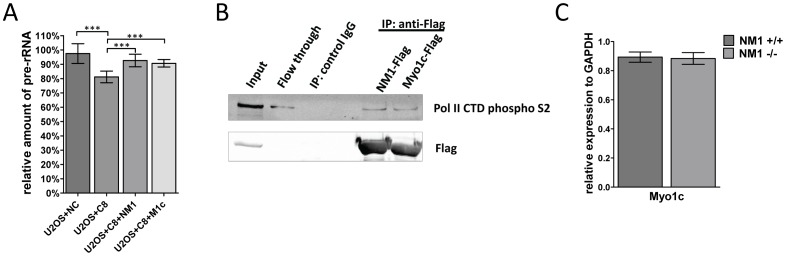
Myo1C is able to function in Pol I and Pol II transcription without changes in its expression level. (**A**) The level of nascent rRNA was decreased to 80% of WT levels in NM1/Myo1c knock-down cells (U2OS+C8). An overexpression of mouse NM1 (U2OS+C8+NM1) or Myo1c (U2OS+C8+M1c) resistant to shRNA causes restoration of Pol I transcription to almost endogenous levels. As a negative control were used U2OS cells with transduced empty pLKO1.1 vector (U2OS+NC). (**B**) Both NM1-Flag and Myo1c-Flag interact with Pol II. Extracts from cells overexpressing NM1-Flag and Myo1c-Flag were co-immunoprecipitated with Flag antibody and control IgG. Immunoprecipitates were analyzed by western blotting with antibodies against Flag, Pol II CTD subunit and Myo1c (tail domain recognizing both NM1 and Myo1c). (**C**) NM1 knock-out does not cause compensatory changes in expression of Myo1c. Expression of Myo1c was measured by RT-qPCR and compared relative to GAPDH expression. Data are presented as mean +/− SD. *** p<0.001.

Since the cellular distribution and expression levels of NM1 and Myo1c are similar, we also explored the possible functional similarities between these isoforms. Previous experiments showed that NM1 depletion decreased RNA polymerase I transcription rate [16]. To test whether Myo1c is able to functionally substitute NM1 in RNA pol I transcription, we used shRNA mediated depletion of NM1/Myo1c in U2OS cells. After decreasing Pol I transcription rate, we were able to reconstitute the phenotype by overexpressing mouse Myo1c that was resistant to the RNAi. Overepression of Myo1c similarly to restored Pol I transcription rate back to almost endogenous level in RNAi depleted cells (**Fig. 5A**). Moreover, NM1 was found to be a component of Pol II transcription machinery and was found to bind to Pol II [6]. We already showed that Myo1c appears to have the same function in Pol I transcription as NM1. To prove if Myo1C would also mimic the function of NM1 with Pol II, we explored possible interacting partners which would belong to molecular components of Pol II transcriptional complex. When immunoprecipitating NM1 or Myo1c proteins tagged with Flag-tag, we succeeded in co-precipitating the CTD domain of Pol II (**Fig. 5B**). This together with previous results showing no significant differences in cell proliferation and viability in NM1 KO cells suggests a role for Myo1c also in Pol II transcription.

### Knock-out of NM1 protein has no effect on Myo1c expression

In a previous experiment, we claimed that Myo1c seems to be able to functionally substitute NM1 in Pol I transcription. To test whether NM1 knock-out leads to a compensatory overexpression of Myo1C, we isolated RNA from NM1 WT and KO skin fibroblasts in the exponential phase of growth, and measured Myo1c expression by RT-qPCR. All experiments were done in triplicates, and we used primers specifically amplifying just the Myo1c coding region. However, the level of expression of Myo1c in both cell lines was comparable (**Fig. 5C**). This result suggests that the amount of Myo1C in the nucleus is sufficient to fulfill the roles in Pol I and Pol II transcription by itself.

Taken together, Myo1c is able to compensate for the loss of NM1. Because the amount of Myo1c is equal to NM1 and there is no compensatory overexpression of Myo1c in NM1 KO cells, we suggest that both isoforms are redundant to each other and can have same functions in Pol I transcription. Moreover, Myo1C is able to bind to CTD domain of Pol II suggesting that these proteins also have identical roles in Pol II transcription.

## Discussion

Nuclear myosin I (Myo1c isoform B), the first molecular motor protein which has been discovered in the cell nucleus, has brought a revolution into the myosin field. In comparison to conventional class I myosins which were described in different functions related to plasma membrane and cytoskeleton, endo- and exo- cytosis, cell motility and cell spreading [35], NM1 was found to be directly involved in processes of RNA Pol I and RNA Pol II transcription, chromatin remodeling and chromosome movements in the cell nucleus. Since its discovery in 1997, 5 others myosins have been observed in the cell nucleus (myosin VI, Va, Vb, XVIb, and XVIIIb); [36]. Recently, a new isoform of human myosin 1c protein-isoform A-was discovered and found to localize to the nucleus. Similar to NM1, this isoform contains a unique N-terminal peptide sequence and co-localizes with RNA polymerase in the nucleoplasm. However, unlike NM1, upon exposure to inhibitors of RNA polymerase II transcription, the newly identified isoform translocates to nuclear speckles. Furthermore, in contrast to NM1, this new isoform is absent from nucleoli and does not co-localize with RNA polymerase I [7].

We showed previously that the “cytoplasmic” Myo1c protein (isoform C) is also able to localize to the nucleus in the same manner as the other isoforms via NLS signal localized in the middle part of molecule [24]. To test the influence of the specific N-terminal 16 amino acids on NM1 functions, we prepared knock-out mice lacking the exon -1 which contains the NM1 start codon. The resulting mRNA only contains the downstream start of translation which gives rise to Myo1c isoform C protein. Surprisingly, knock-out mice were without any obvious phenotype, and proliferation assays together with RT-qPCR of 45S pre-rRNA did not show any differences or aberrance in RNA Pol I and Pol II transcription. This could be explained by compensatory expression of other myosin motor protein, acting in transcription, or by the fact that the overall level of myosin motor proteins in the nucleus is redundant and therefore a depletion of one isoform does not affect transcription. To test these hypotheses, we depleted Myo1c isoforms from U2OS cells by shRNA in order to decrease Pol I transcription. We then measured the reconstitution of transcription after overexpression of mouse Myo1c that was unsusceptible to the RNAi. Exogenously expressed Myo1c was able to restore the Pol I transcription rate to nearly the endogenous level, apparently compensating for the loss of NM1. Moreover, we found that the ratio between both isoforms in the nucleus of WT cells is equal and there is no compensatory change in expression of Myo1c in NM1 KO cells, suggesting that Myo1c is transported to the nucleus normally and its amount in nucleus is sufficient to fulfill myosin functions in transcription by itself. In conclusion, the two different isoforms (B and C) in the nucleus seem to be redundant for effective Pol I transcription. Moreover, Myo1c is able to bind to CTD domain of Pol II suggesting its same role also in Pol II transcription.

However, as mentioned above, the third Myo1c isoform-isoform A-carrying different and longer N-terminal extension has slightly different cytoplasmic and nuclear localization in comparison to NM1 [7], and we also noted previously that localization pattern of exogenously expressed NM1 and Myo1c do not fully overlap [24]. This could suggest different roles for each isoform, dependent on their N-terminal extensions, but most probably without affecting the general process of transcription by Pol I or Pol II. Because we did not detect changes in Pol I transcription in NM1 KO cells, it is possible that different N-terminal extensions will have a role in a fine tuning of myosin functions under special conditions or in specialized cell types. It would be therefore interesting to explore these three isoforms under various stress conditions (i.e. UV, starvation, heat shocks) and in different tissues and cell lines. However, to come to a definitive conclusion, conditional knock-out mouse and derived cell lines lacking all three isoforms are needed.

Studies of NM1 function in tissue and at a whole body level did not show any obvious deviations in comparison to WT mice. However, we discovered minor differences in bone mineral density of KO mice, and discovered several genes with changed expression profiles in NM1 KO mice in relation with lipid metabolism in adipocytes (data not shown). This could relate bone metabolism with insulin signaling and energy metabolism as was described previously [37], [38], but the function of NM1 in this process is not known and has to be further studied. However, it was shown that Myo1c is highly expressed in adipocytes and is responsible for movements of GLUT-4 containing vesicles upon insulin stimulus [13]. Therefore we cannot exclude the possibility that NM1 is also playing some role in these processes, but we suppose that its function would be related to the cytoplasmic rather than to nuclear functions. However, to support this speculation, future biochemical analysis of pathways of the insulin signaling in NM1 KO mice are required.

Experiments monitoring physiological changes included also hematology analysis which revealed mild hyperchromic macrocytosis of red blood cells in mutant males characterized by larger red blood cells containing a higher amount of hemoglobin. This phenotype suggest an impaired cell division during erythropoiesis [34]. Although, in cell culture cell function of the cell cycle machinery did not show a detectable effect, rate limitation concerning proliferation might occur in vivo on a high level due to decreased amounts of Myo1c being available in the nucleus. This could results in mild detectable effects in hematopoiesis since hematopoietic tissue has an extremely high proliferation rate. This would be comparable to the effect of some missense mutations in ribosomal protein S19, which can be associated with macrocytosis as the only or one of some mild symptoms in the heterozygous state in men and mice [39], [40]. Another possible explanation is that red blood cells in NM1 knock-out mice have partially impaired linkage between plasma membrane and cytoskeleton, and therefore they could show increased mean corpuscular volume. This suggestion is in agreement with previous data on different members of Myosin I family, which were shown to work as a dynamic link between plasma membrane and cytoskeleton [41]. In particular; class I myosins mediate membrane/cytoskeleton adhesion and thus they make major contribution to membrane tension. Their study showed that class I myosins directly control the mechanical properties of the cell membrane and are master regulators of cellular events involving membrane deformation. Microarray analysis from NM1 KO mice and co-immunoprecipitation experiments, which identified mostly cytoplasmic and membranous proteins link NM1 to similar processes, described for the other members of Myosin I family (Kalendova and Venit, in preparation). However, results mentioned above suggest some new role of NM1 on the cell membrane and in cytoplasmic shuttling but not in nucleus/nucleolus.

In conclusion, we prepared mice lacking functional nuclear myosin 1 protein, which has been described as a key player in Pol I and Pol II transcription. Knock-out mice do not show any obvious pathological phenotype, which can be explained by functioning of “cytoplasmic” Myo1c isoform in nucleus in general process of transcription. Both proteins have nearly equal expression levels and distribution in the cell and a knock-out of NM1 does not cause any compensatory changes in Myo1c expression. Therefore, we suggest that both isoforms are interchangeable and redundant with each other in the cell nucleus. This data raises additional interesting questions: What is the functional significance of the different N-terminal regions of the myosin molecules in nuclear and cytoplasmic processes? What are the functions of NM1 in the cytoplasm and on plasma membrane? What is the relationship between these isoforms in the different cell types or tissues? Obviously, some further investigation and preparation of new tools will be needed to understand the interplays of Myosin 1c isoforms.
